# *Sarcocystis* sp. infection (Apicomplexa: Sarcocystidae) in invasive California kingsnake *Lampropeltis californiae* (Serpentes: Colubridae) in Gran Canaria

**DOI:** 10.1017/S0031182022000841

**Published:** 2022-09

**Authors:** Kevin M. Santana-Hernández, Kristýna Javorská, Eligia Rodríguez-Ponce, Barbora Fecková, Jan Šlapeta, David Modrý

**Affiliations:** 1 Department of Animal Pathology, Faculty of Veterinary Sciences, University of Las Palmas de Gran Canaria, Arucas, Spain; 2 Department of Veterinary Sciences, Faculty of Agrobiology, Food and Natural Resources/CINeZ, Czech University of Life Sciences Prague, Prague, Czech Republic; 3 Department of Pathology and Parasitology, Faculty of Veterinary Medicine, University of Veterinary and Pharmaceutical Sciences, Brno, Czech Republic; 4 Sydney School of Veterinary Science, Faculty of Science, The University of Sydney, New South Wales, Australia; 5 Department of Botany and Zoology, Faculty of Science, Masaryk University, Brno, Czech Republic; 6 Institute of Parasitology, Biology Centre CAS, České Budějovice, Czech Republic

**Keywords:** Canary Islands, invasive species, *Lampropeltis*, *Sarcocystis*

## Abstract

Invasive species pose a threat not only to biodiversity because they displace or compete with native fauna, but also because of the pathogens they can host. The Canary Islands are an Atlantic biodiversity hotspot threatened by increasing numbers of invasive species, including the California kingsnake *Lampropeltis californiae*, which was recently introduced to Gran Canaria. Seventy-seven snakes were examined for gastrointestinal parasites in 2019–2020. Sporocysts of *Sarcocystis* sp. were detected in 10 of them; detection of gamogonia stages in histological sections of 3 snakes confirmed the snake as a definitive host. Partial ssrDNA was amplified using SarcoFext/SarcoRext primers; an additional sequence of *Sarcocystis* was obtained from the tail muscle of the endemic Gran Canaria giant lizard *Gallotia stehlini* for a comparison. Identical ssrDNA sequences of unknown *Sarcocystis* sp. were obtained from 5 different snakes. Phylogenetic analysis showed that *Sarcocystis* sp. isolated from invasive California kingsnakes is unrelated to *Sarcocystis* provisionally considered *S. stehlini* from the endemic lizard. The dixenous coccidia are rarely reported to invade new predator–prey systems. However, the present data suggest that previously unknown *Sarcocystis* sp. is circulating among invasive snakes and as yet unknown vertebrate intermediate hosts, with undetermined consequences for the Gran Canaria ecosystem.

## Graphical abstract

**Figure:**
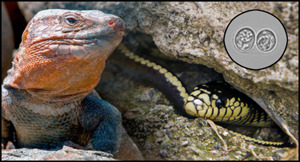

## Introduction

The Canary Islands are characterized by a great diversity of endemic reptiles, which constitute a large part of the terrestrial vertebrate fauna of the archipelago (Arechavaleta *et al*., 2010). Like other volcanic islands, the Canary Islands were colonized by reptiles through a series of long-distance dispersal events, in this case from mainland Africa (Illera *et al*., 2016). Three saurian genera, *Gallotia*, *Chalcides* and *Tarentola*, belonging to the families Lacertidae, Scincidae and Geckonidae, respectively, have diversified into 15 extant species *via* processes of adaptive radiation (Brown and Pestano, 1998; Carranza *et al*., 2000; Cox *et al*., 2010).

**Fig. 1.**
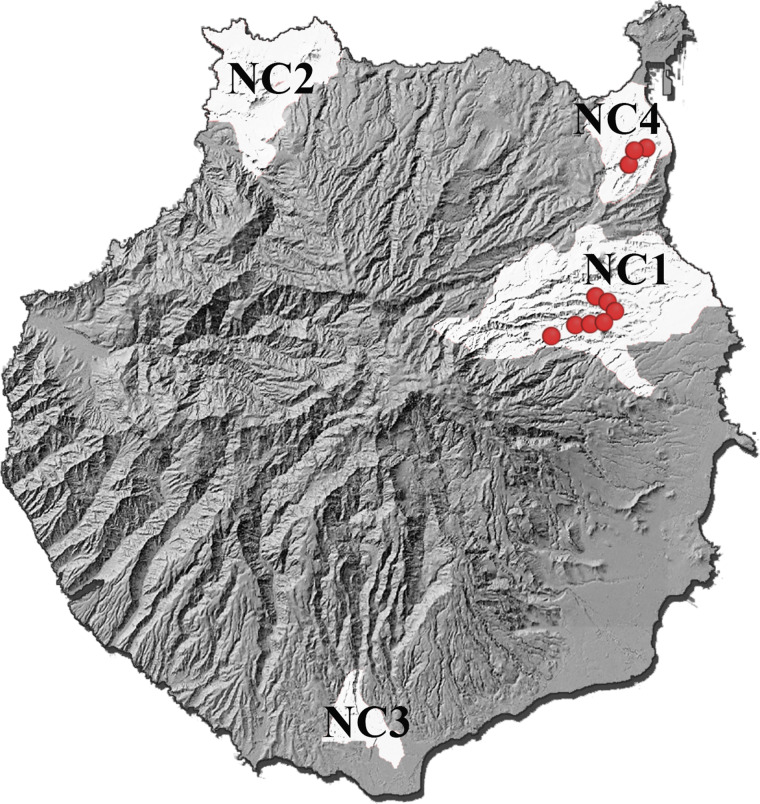
A map of Gran Canaria island showing 4 populations of *Lampropeltis californiae* as white-shaded areas (NC1–NC4); the place of collection of 10 snakes positive for *Sarcocystis* sp. based on coproscopic examination marked by red dots. NC1: Main nucleus. NC2: Secondary nucleus. NC3: Third nucleus. NC4: Fourth nucleus.

Invasive species threaten biodiversity not only through predation or competition with native fauna, but also through the pathogens they can transmit. Emerging infectious diseases are an important phenomenon underlying biological invasions, and oceanic islands are more vulnerable to biological invasions than continental ecosystems (Gurevitch and Padilla, 2004; Carroll, 2007). One invasive species in the Canary Islands stands out among the others and probably represents the greatest threat to the endemic vertebrates of the island of Gran Canaria: the California kingsnake, *Lampropeltis californiae* Blainville, 1835. The first observations of this North American colubrid were reported in southeastern Gran Canaria in 1998 (Pether and Mateo, 2007), but the eradication programme was not launched until 2007. Surveys in the following years revealed a large population that had expanded to the adjacent central and higher areas of the island, forming the so-called ‘main nucleus’ (NC1, Fig. 1). In 2010, the presence of a secondary nucleus (NC2) was confirmed in the northwest of the island (Gallo-Barneto *et al*., 2016). Subsequent phenotypic and molecular studies suggested that the kingsnakes in these 2 nuclei originated from 2 different introductions (Monzón-Argüello *et al*., 2015). Despite the eradication programme, the establishment of a small population was also confirmed in the south of the island (the third nucleus, NC3) and in the northeast (the fourth nucleus, NC4). In all cases, populations of *L. californiae* occur in habitats that are considered Special Conservation Area (BOC no. 60, 15 May 2000) and are inhabited by a variety of endemic species.

Dietary studies have confirmed the high proportion of endemic reptiles in the diet of snakes (69%), highlighting the Gran Canaria giant lizard *Gallotia stehlini* as the main component (43%), followed by the Gran Canaria skink *Chalcides sexlineatus* (23%) and the Boettger's wall gecko *Tarentola boettgeri* (3%) (Cabrera-Pérez *et al*., 2012; Monzón-Argüello *et al*., 2015). Small mammals (29.5%) and birds (1.5%) have also been detected in the digestive system of snakes. In addition to direct effects by predation on endemic fauna, possible co-invasive metazoan parasites infecting the snake have been detected, as well as ‘reverse’ infection with larval stages of parasites of feral cats and other vertebrates (Santana-Hernández *et al*., 2021).

Well-described evolutionary processes and the diversity of reptiles make the Canary Islands an interesting model site that has stimulated several parasitological studies involving both protists and helminths (Roca *et al*., 2012; Jorge *et al*., 2018). Among protistan parasites with heteroxenous life cycles, 2 groups have recently gained attention – haemogregarines and *Sarcocystis* (e.g. Matuschka and Bannert, 1989; Bannert, 1992; Tomé *et al*., 2018, 2019; Illera Cobo and Perera, 2020). The latter genus is characterized by a 2-host life cycle, that typically involves predators and their prey (in the case of reptiles, commonly colubrid snakes and lacertid or geckonid lizards). However, *Sarcocystis* species described in Canary Island lizards of the genus *Gallotia* are particularly adapted to transmission in an environment where there are no predators. In their life cycle (usually referred to as dihomoxenous), merogony and gamogony occur in the same host species (in this case *Gallotia* lizards), which serves alternately as the final and intermediate host. Infection is transmitted among lizards *via* sporocysts in feces and cannibalism (Matuschka and Bannert, 1987, 1989).

The introduction of an invasive snake predator into a Macaronesian ecosystem inhabited by endemic saurian reptiles provides an unintended ‘experimental’ system to evaluate the host specificity of *Sarcocystis* spp. in, and its potential interactions with, the invasive kingsnake *L. californiae*.

## Material and methods

### Reptiles collected, examination

In the period 2019–2020, samples of California kingsnakes were analysed. The snakes were captured by the staff of Gestión y planeamiento territorial y ambiental (GesPlan) manually and using box-traps in the framework of the eradication project (https://www.gesplan.es/content/orden-33620-que-modifica-la-n%C2%BA-12419-ejecucion-plan-post-life-lampropeltis-y-actuaciones-del). The locality data, colour pattern, sex, length and weight of each animal (Table 1) were collected. Captured snakes were euthanized with intracardiac premedication of mixed ketamine and medetomidine (5–10 mg kg^−1^ and 0.15–0.5 mg kg^−1^), and a lethal dose (0.5–1 mL kg^−1^) of pentobarbital by the veterinarians from the eradication programme.

**Table 1.** Distribution of snakes regarding nuclei, the municipalities which encompasses and positive/negative to *Sarcocystis* sp.

Nuclei (snakes examined)	Municipality	Positive (males/females)	Total (males/females)
**1st (61)**	Telde	6 (4/2)	52 (20/32)
	Valsequillo	1 (1/0)	8 (6/2)
	Santa Brígida	0	1 (1/0)
**2nd (3)**	Gáldar	0	2 (0/2)
	Agaete	0	1 (0/1)
**3rd (5)**	San Bartolomé	0	5 (0/5)
**4th (8)**	Las Palmas de Gran Canaria	3 (2/1)	8 (4/4)
**Total**	8 Municipalities	10 (7/3)	77(31/46)

The feces collected at the time of euthanasia was kept in sterile containers, transported in a refrigerator (3 °C) and examined within the following 12 h in the Parasitology Laboratory of the Faculty of Veterinary Sciences of the University of Las Palmas de Gran Canaria. The fecal samples were examined microscopically after centrifugal flotation with Sheather's sugar solution (Zajac and Conboy, 2012), using a Nikon Eclipse Ni-U microscope with a Nikon Ds-Fi2 camera.

The small intestines of euthanized snakes were extracted and fixed in ethanol and 10% buffered formalin (Farris *et al*., 2013). After coproscopic examination, the small intestine samples that were positive for *Sarcocystis* sp. (i.e. in which sporocysts/ocysts were detected by microscopy) were further processed. Specimens preserved in formalin were processed for standard histopathological examination and stained with haematoxylin and eosin (H&E). Sections were examined with Olympus BX53 light microscope and photographed with Olympus DP73 camera and Olympus Dimension CellSens imaging software. As comparative material, 2 Gran Canaria giant lizards *Gallotia stehlini* Schenkel, 1901 were received dead from the Wildlife Recovery Centre of Tafira, 1 from the municipality of San Mateo and 1 from Arucas. The tail muscles were microscopically examined as squash preparations for the presence of *Sarcocystis* tissue cysts and preserved in ethanol.

### DNA isolation, PCR and sequencing

A total of 7 snake fecal samples, 3 snake gut tissue samples, and 2 lizard tail muscle tissue samples, all stored in ethanol, were used for DNA isolation. Genomic DNA was isolated using the GeneAll Exgene™ Stool DNA mini kit for fecal samples and the NucleoSpin^®^ Tissue kit (Macherey-Nagel) for tissue samples according to the manufacturer's instructions. The ssrRNA gene fragment was amplified using SarcoFext (5′-GGTGATTCATAGTAACCGAACG-3′)/SarcoRext (5′-GATTTCTCATAAGGTGCAGGAG-3′) primers (Moré *et al*., 2013). To increase PCR sensitivity in isolates number 8 and 14, the following nested-PCR protocol was developed. A total volume of 25 *μ*L PCR reaction mixture consisted of 12.5 *μ*L PRCBIO Taq Mix Red Mastermix (PCR Biosystems Ltd.), 8.5 *μ*L PCR H_2_O, 1 *μ*L of each primer and 2 *μ*L extracted DNA. Amplification began with an initial denaturation at 95 °C for 1 min, followed by 40 cycles of 95 °C (15 s), 57 °C (15 s), and 72 °C (15 s), and ended with a final elongation at 72 °C for 5 min. The primers used in the first run were SarcoFext/SarcoRext (see above) and in the second run SarcoFint (5′-CGCAAATTACCCAATCCTGA-3′)/SarcoRint (5′-ATCGTCTTCGAGCCCCTAAC-3′) (Moré *et al*., 2013). PCR products positive in gel electrophoresis (1% agarose gel) were purified using the Gel/PCR DNA Fragments Extraction Kit (Geneaid Biotech Ltd.) and sequenced by Macrogen Europe B.V.

### Phylogenetic and statistical analyses

The sequences obtained were processed using Geneious Prime^®^ 2020.2.5 software (Biomatters Ltd.). The following sequences from GenBank (accession numbers in parentheses) were selected for comparison in phylogenetic analysis: *Sarcocystis* sp. (KX453662), *S. lacertae* (AY015113), *S. gallotiae* (AY015112), *S. muris* (KC877996), *S. rodentifelis* (AY015111), *Frenkelia microti* (AF009244), *F. glareoli* (AF009245), *S. jamaicensis* (KY994649), *S. speeri* (KT207459), *S. neurona* (U07812), *S. ramphastosi* (EU263366), *S. falcatula* (MH626537), *S. lari* (MF946588), *S. rileyi* (KJ396583), *Sarcocystis* sp. (KX833709) and *Sarcocystis* sp. (KX453661). *Sarcocystis* sp. (U97524) and *S. atheridis* (AF120114) as outgroup. Phylogenetic analysis was performed using the maximum likelihood method of the IQ-TREE web server tool (Nguyen *et al*., 2015) with ultrafast bootstrap (Minh *et al*., 2013). The phylogenetic tree was visualized and processed using FigTree v1.4.4 (http://tree.bio.ed.ac.uk/software/figtree/).

Basic statistical analysis was performed using SPSS v. 24.0 (IBM SPSS Corp., Chicago, Illinois, USA). The map was created using My map app by Google and GIMP 2.10 (GNU Image Manipulation program by Spencer Kimball, Peter Mattis and GIMP staff).

## Results

A total of 77 kingsnakes and 2 lizards were examined for the presence of *Sarcocystis* sp. during the study period. Snakes from 7 different localities comprising the 4 nuclei were examined (Fig. 1). Seventy-eight per cent of the snakes were from the main nucleus. Only individuals from the main nucleus (Telde and Valsequillo) and the fourth nucleus (Las Palmas de Gran Canaria) were positive for the presence of *Sarcocystis* sp. in feces (Table 1). The average biometric parameters of the snakes studied are summarized in Table 2. A subtle difference was observed between positive (shorter and lighter) and negative animals.

**Table 2.** Biometrical parameters of positive and negative snakes to *Sarcocystis* sp.

		Positive	Negative	Total
Males	Length	830.0 ± 214.0	967.6 ± 176.0	936.5 ± 190.6
	Weight	203.3 ± 121.8	289.7 ± 138.1	270 ± 137.6
	*n*	7	24	31
Females	Length	853.3 ± 134.3	877.1 ± 154.7	875.6 ± 152.3
	Weight	198.3 ± 134.3	242.6 ± 142.7	239.7 ± 136.7
	*n*	3	43	46
Total	Length	837.0 ± 186.2	909.5 ± 167.2	900.1 ± 170.2
	Weight	201.8 ± 108.4	259.5 ± 140.0	252.0 ± 137.0
	*n*	10	67	77

All the measurements for length and weight are in millimetres and grams.

### Coproscopy and histopathology

**Fig. 2.**
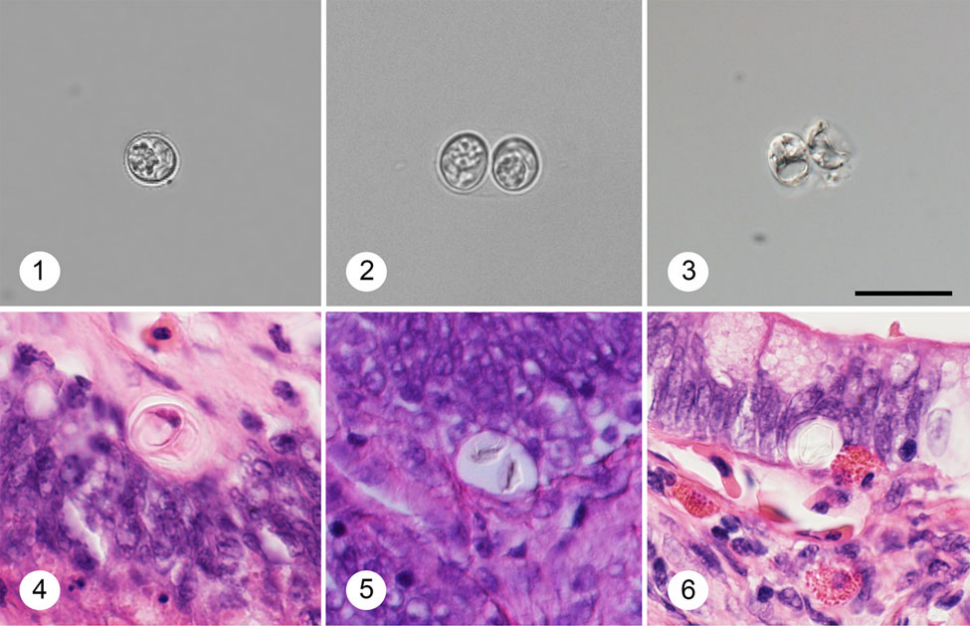
Developmetal stages of *Sarcocystis* sp. in feces after flotation (1–3) and in intestinal tissue (4 and 5) stained with H&E. 1: Isolated sporocyst with apparent sporocyst residuum; 2: intact oocyst with apparent oocyst wall; 3: oocysts deformed by preservation; 4: *in situ* sporulated oocyst with apparent sporozoites; 5 and 6: cross-section through oocyst in parasitophorous vacuole showing the sporocyst wall. All figures in same scale, scale bar = 20 *μ*m, figs 3 and 5 photographed with Nomarski differential contrast.

Oocysts and sporocysts of *Sarcocystis* sp. were detected by microscopy in the feces of 10 of the 77 snakes (Fig. 2). Liberated sporocysts (Fig. 2.1) were more common, although both types of exogenous stages were present in very low numbers. Sporocysts were broadly ellipsoidal, 11.5–12.5 × 9.7–10.3 (*n* = 5), with granular sporocyst residuum and 4 readily identifiable sporozoites.

Scattered developmental stages of gamogony of *Sarcocystis* sp. were observed in small intestinal sections of 3 California kingsnakes. The *in situ* sporulated oocysts were singly localized between enterocytes at the border of the lamina propria mucosae (Figs 2.4–2.6). No other developmental stages were seen.

### Sequencing and phylogenetic analyses

**Fig. 3.**
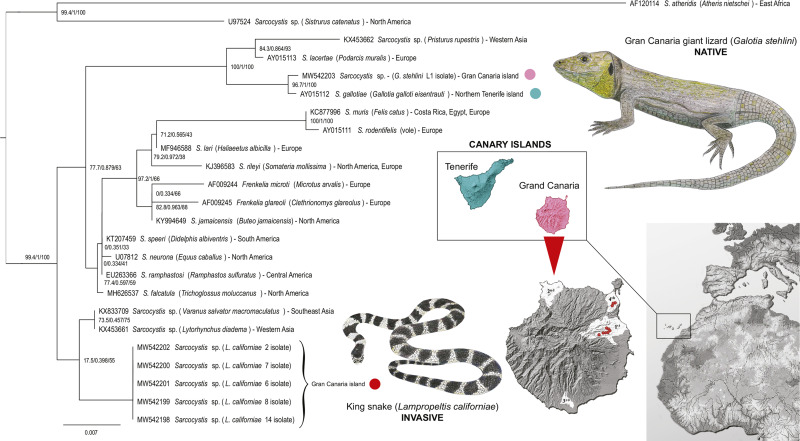
Phylogenetic analysis of the partial ssrDNA sequences showing position of the 2 taxa discussed. The sequences used for comparison were chosen from a phylogenetic tree containing all GenBank accessible ssrDNA sequences of *Sarcocystis* spp. (data not shown) and the current tree consists of the sequences most related to those extracted from *L. californiae* and *G. stehlini*, rooted on *S. atheridis* (AF120114) and *Sarcocystis* sp. (U97524). Geographical origin was added to each selected sequence.

Partial ssrRNA gene sequences of *Sarcocystis* sp. were obtained from 5 different California kingsnakes (2 from fecal sediment and 3 from small intestine tissue of snakes that were positive in histopathological sections) and from the tail muscle of a single Gran Canaria giant lizard. The sequences derived from the snakes were identical and 532–811 bp long (short sequences of 532 bp were from isolates processed by nested-PCR); the sequence from the tail muscle of the lizard was 785 bp long. The sequences obtained are deposited in GenBank under the following accession numbers: MW542198–MW542202 (from snakes) and MW542203 (from a lizard). The sequences obtained from the snakes and the lizard had 95.9% similarity. Phylogenetic analysis revealed that *Sarcocystis* sp. from the California kingsnakes in this study was most closely related to *Sarcocystis* sp. isolated from the crowned leaf-nosed snake (*Lytorhynchus diadema* – KX453661) and the common water monitor (*Varanus salvator macromaculatus* – KX833709), with pairwise similarity of 99.7% and 99.3%, respectively. *Sarcocystis* sp. isolated from the Gran Canaria giant lizard branched with *S. gallotiae* (AY015112) from *G. galloti* from Tenerife, with 99.7% similarity (Fig. 3).

## Discussion

The finding of a *Sarcocystis* species in a population of invasive North American colubrid snakes recently introduced to Gran Canaria was unexpected, as the dixenous coccidia rarely invade new predator–prey systems. All the positive animals were found at the east nuclei of the island (main nucleus = 7/61 and fourth nucleus = 3/8). However, considering the huge difference of sample size between the nuclei no further statistical analyses were considered.

Three *Sarcocystis* spp. were previously described in endemic *Gallotia* lizards of Canary Islands, all of which have a dihomoxenous life cycle (Matuschka and Bannert, 1987, 1989; Bannert, 1992). In a previous phylogenetic study, *Sarcocystis gallotiae* was found to branch with Old World *Sarcocystis* species with a snake–lizard life cycle (Šlapeta *et al*., 2001). Since *Gallotia* lizards evolved from North African ancestors (Cox *et al*., 2010; Illera *et al*., 2016), it is likely that the ancestral *Sarcocystis* had cycled between African lacertids and snakes. The DNA sequences of *Sarcocystis* that we obtained from invasive snakes and *Sarcocystis* from an endemic lizard *G. stehlini* differed significantly and branched into distant clades. On the contrary, the lizard-derived sequence was very close to *S. gallotiae* (>99 identity), which was previously isolated from *G. galloti* from Tenerife (Šlapeta *et al*., 2001).

It is very likely that the *Sarcocystis* from the California kingsnakes described here is a snake-specific species that probably invaded the Macaronesian ecosystem along with its snake host. *Sarcocystis* species using reptiles as definitive hosts exhibit host specificity restricted to their host genus or family and are most likely unable to switch between hosts from different reptilian orders (Box and Smith, 1982; Lindsay *et al*., 1992).

Small mammals (such as rodents) are the most common intermediate hosts for *Sarcocystis* species that have a snake as a definitive host. However, some *Sarcocystis* spp. have been described to cycle between snakes and saurian reptilian hosts (Volf *et al*., 1999; Modrý *et al*., 2000). The intermediate host of *Sarcocystis* sp. found in the kingsnakes of Gran Canaria is still unknown and further research should be directed towards the study of native (snakes, skins, geckos, passerine birds) or invasive (mice and rats) vertebrates, which inhabit in all nuclei.

The exact origin of the presumably invasive *Sarcocystis* species remains unknown, as does the origin of the introduced California kingsnakes themselves. Maintenance of *Sarcocystis* infection in captive-bred snakes is unlikely because they cannot sustain a 2 host life cycles. On the contrary, the presence of patent *Sarcocystis* spp. infection in captive-bred snakes strongly suggests that they are wild-caught (Moré *et al*., 2014). In this sense, the presence of *Sarcocystis* sp. in invasive kingsnakes in Gran Canaria suggests that the population was established rather by individuals introduced from wild populations in the southwestern United States of northern Mexico than by captive-borne pet snakes.

Clarification of the life cycle of the snake sarcosporidian found in the Gran Canaria is necessary to answer questions related to possible impacts on the endangered endemic vertebrate fauna of Macaronesia.

## Data Availability

Data are available under reasonable requests.

## References

[ref1] Arechavaleta M, Rodríguez S, Zurita N and García A (2010) Lista de especies silvestres de Canarias (hongos, plantas y animales terrestres) 2009. Canary Islands, Spain: Gobierno de canarias, 579pp. https://www.researchgate.net/publication/261124878_Listas_de_Especies_Silvestres_de_Canarias_Hongos_Plantas_y_Animales_Terrestres_2009

[ref2] Bannert B (1992) *Sarcocystis simonyi* sp. nov. (Apicomplexa: Sarcocystidae) from the endangered Hierro giant lizard *Gallotia simonyi* (Reptilia: Lacertidae). Parasitology Research 78, 142–145.

[ref3] Box ED and Smith JH (1982) The intermediate host spectrum in a *Sarcocystis* species of birds. Journal of Parasitology 68, 668–673. 6811715

[ref4] Brown RP and Pestano J (1998) Phylogeography of skinks (*Chalcides*) in the Canary Islands inferred from mitochondrial DNA sequences. Molecular Ecology 7, 1183–1191. 9734075 10.1046/j.1365-294x.1998.00442.x

[ref5] Cabrera-Pérez MÁ, Gallo-Barneto R, Esteve I, Patiño-Martínez C and López-Jurado LF (2012) The management and control of the California kingsnake in Gran Canaria (Canary Islands): project LIFE+ *Lampropeltis*. Aliens: The Invasive Species Bulletin 32, 20–28.

[ref6] Carranza S, Arnold EN, Mateo JA and López-Jurado LF (2000) Long-distance colonization and radiation in gekkonid lizards, *Tarentola* (Reptilia: Gekkonidae), revealed by mitochondrial DNA sequences. Proceedings of the Royal Society of London B 267, 637–649. 10.1098/rspb.2000.1050 PMC1690580 10821607

[ref7] Carroll SP (2007). Natives adapting to invasive species: ecology, genes, and the sustainability of conservation. Ecological Research 22, 892–901.

[ref8] Cox SC, Carranza S and Brown RP (2010). Divergence times and colonization of the Canary Islands by *Gallotia* lizards. Molecular Phylogenetics and Evolution 56, 747–757. 20307675 10.1016/j.ympev.2010.03.020

[ref9] Farris S, Squires M, Ridgley F, Lavergne E, Serota MW and Mazzotti FJ (2013) Necropsies of Reptiles: Recommendations and Techniques for Examining Invasive Species. Glainesville, FL, USA: Electronic DataInformation Source, Institute of Food and Agricultural Sciences, University of Florida.

[ref10] Gallo-Barneto R, Cabrera-Pérez MÁ, Peña-Estevez MÁ, Patiño-Martinez C and Monzón-Argüello C (2016) The California kingsnake. An intruder in the garden of the Hesperides. InDiferente 22, 126–141.

[ref11] Gurevitch J and Padilla DK (2004) Are invasive species a major cause of extinctions? Trends in Ecology and Evolution 19, 470–618. 16701309 10.1016/j.tree.2004.07.005

[ref12] Illera JC, Spurgin LG, Rodriguez-Exposito E, Nogales M and Rando JC (2016) What are we learning about speciation and extinction from the Canary Islands? Ardeola 63, 5–23.

[ref13] Illera Cobo JC and Perera A (2020) Where are we in the host-parasite relationships of native land vertebrates in Macaronesia? Ecosistemas 29, 1–10. doi: 10.7818/ECOS.1971

[ref14] Jorge F, Perera A, Poulin R, Roca V and Carretero MA (2018) Getting there and around: host range oscillations during colonization of the Canary Islands by the parasitic nematode *Spauligodon*. Molecular Ecology 27, 533–549. 29219226 10.1111/mec.14458

[ref15] Lindsay DS, Upton SJ, Blagburn BL, Toiviokinnucan M, Dubey JP, McAllister CT and Trauth SE (1992) Demonstration *that Sarcocystis montanaensis* has a speckled kingsnake–prairie vole life cycle. Journal of the Helminthological Society of Washington 59, 9–15.

[ref16] Matuschka FR and Bannert B (1987) Cannibalism and autotomy as predator-prey relationship for monoxenous Sarcosporidia. Parasitology Research 74, 88–93. 3125543 10.1007/BF00534938

[ref17] Matuschka FR and Bannert B (1989) Recognition of cyclic transmission of *Sarcocystis stehlinii* n. sp. in the Gran Canarian giant lizard. The Journal of Parasitology 75, 383–387. 2498493

[ref18] Minh BQ, Minh ATN and von Haeseler A (2013) Ultrafast approximation for phylogenetic bootstrap. Molecular Biology and Evolution 30, 1188–1195. 23418397 10.1093/molbev/mst024 PMC3670741

[ref19] Modrý D, Koudela B and Šlapeta JR (2000) *Sarcocystis stenodactylicolubris* n. sp., a new sarcosporidian coccidium with a snake-gecko heteroxenous life cycle. Parasite (Paris, France) 7, 201–207. 11031756 10.1051/parasite/2000073201

[ref20] Monzón-Argüello C, Patiño-Martínez C, Christiansen F, Gallo-Barneto R, Cabrera-Pérez MÁ, Peña-Estévez MÁ, López-Jurado LF and Lee PLM (2015) Snakes on an island: independent introductions have different potentials for invasion. Conservation Genetics 16, 1225–1241.

[ref21] Moré G, Schares S, Maksimov A, Conraths FJ, Venturini MC and Schares G (2013) Development of a multiplex real time PCR to differentiate *Sarcocystis* spp. affecting cattle. Veterinary Parasitology 197, 85–94. 23680541 10.1016/j.vetpar.2013.04.024

[ref22] Moré G, Pantchev N, Herrmann DC, Vrhovec MG, Öfner S, Conraths FJ and Schares G (2014) Molecular identification of *Sarcocystis* spp. helped to define the origin of green pythons (*Morelia viridis*) confiscated in Germany. Parasitology 141, 646–651. 24476633 10.1017/S0031182013001960

[ref23] Nguyen LT, Schmidt HA, von Haeseler A and Minh BQ (2015) IQ-TREE: a fast and effective stochastic algorithm for estimating maximum-likelihood phylogenies. Molecular Biology and Evolution 32, 268–274. 25371430 10.1093/molbev/msu300 PMC4271533

[ref24] Pether J and Mateo JA (2007) La culebra real (*Lampropeltis getulus*) en Gran Canaria, otro caso preocupante de reptil introducido en el Archipiélago Canario. Boletín de La Asociación Herpétologica Española 18, 20–23.

[ref25] Roca V, Jorge F and Carretero MA (2012) Synopsis of the helminth communities of the lacertid lizards from Baleartic and Canary Islands. Basic and Applied Herpethology 26, 107–116.

[ref26] Santana-Hernández K, Orós J, Priestnall S, Monzón-Argüello C and Rodríguez-Ponce E (2021) Parasitological findings in the invasive California kingsnake (*Lampropeltis californiae*) in Gran Canaria, Spain. Parasitology 148, 1345–1352. 34096484 10.1017/S0031182021000871 PMC8383276

[ref27] Šlapeta JR, Modrý D, Votýpka J, Jirků M, Koudela B and Lukeš J (2001) Multiple origin of the dihomoxenous life cycle in sarcosporidia. International Journal for Parasitology 31, 413–417. 11306120 10.1016/s0020-7519(01)00127-8

[ref28] Tomé B, Pereira A, Jorge F, Carretero MA, Harris DJ and Perera A (2018) Along for the ride or missing it altogether: exploring the host specificity and diversity of haemogregarines in the Canary Islands. Parasites & Vectors 11, 1–13. 29554983 10.1186/s13071-018-2760-5 PMC5859493

[ref29] Tomé B, Pereira A, Harris DJ, Carretero MA and Perera A (2019) A paradise for parasites? Seven new haemogregarine species infecting lizards from the Canary Islands. Parasitology 146, 728–739. 30871644 10.1017/S0031182018002160

[ref30] Volf J, Modrý D, Koudela B and Šlapeta JR (1999). Discovery of the life cycle of *Sarcocystis lacertae* Babudieri, 1932 (Apicomplexa: Sarcocystidae), with a species redescription. Folia Parasitologica 46, 257–262.

[ref31] Zajac AM and Conboy GA (2012) Fecal examination for the diagnosis of parasitism. In Veterinary Clinical Parasitology, 8th Edn. Chichester, West Sussex, UK: Wiley-Blackwell, p. 354.

